# Gaze Information Channel in Van Gogh’s Paintings

**DOI:** 10.3390/e22050540

**Published:** 2020-05-12

**Authors:** Qiaohong Hao, Lijing Ma, Mateu Sbert, Miquel Feixas, Jiawan Zhang

**Affiliations:** 1 College of Intelligence and Computing, Tianjin University, Yaguan Road 135, Tianjin 300350, China; qiaohonghao@gmail.com (Q.H.); pt_mary@tju.edu.cn (L.M.); 2 Institute of Informatics and Applications, University of Girona, 17003 Girona, Spain; miquel.feixas@udg.edu

**Keywords:** eye tracking, entropy, gaze information channel, Markov chain, computational aesthetics, Kolmogorov complexity, permutation entropy

## Abstract

This paper uses quantitative eye tracking indicators to analyze the relationship between images of paintings and human viewing. First, we build the eye tracking fixation sequences through areas of interest (AOIs) into an information channel, the gaze channel. Although this channel can be interpreted as a generalization of a first-order Markov chain, we show that the gaze channel is fully independent of this interpretation, and stands even when first-order Markov chain modeling would no longer fit. The entropy of the equilibrium distribution and the conditional entropy of a Markov chain are extended with additional information-theoretic measures, such as joint entropy, mutual information, and conditional entropy of each area of interest. Then, the gaze information channel is applied to analyze a subset of Van Gogh paintings. Van Gogh artworks, classified by art critics into several periods, have been studied under computational aesthetics measures, which include the use of Kolmogorov complexity and permutation entropy. The gaze information channel paradigm allows the information-theoretic measures to analyze both individual gaze behavior and clustered behavior from observers and paintings. Finally, we show that there is a clear correlation between the gaze information channel quantities that come from direct human observation, and the computational aesthetics measures that do not rely on any human observation at all.

## 1. Introduction

The eye is one of the most important organ for human beings to know the external things and transmit information. The eye tracking system can track the trajectory of the eye, thereby obtaining eye movement indicators such as the fixation position, the number of fixations, and fixation duration. By the analysis of eye movement data, the subjective views can be obtained, so that we can expect to improve our ability to measure the individual’s understanding of an image or a scene.

With more and more researchers using eye tracking technology as a research tool, eye tracking is a promising method in academic and industrial research. It has the potential to provide insights into many issues in the visual and cognitive fields: education [1,2,3], medicine [4,5,6,7], assistive technology for people with a variety of debilitating conditions [8,9,10], better interface design [11,12,13], marketing and media [14,15,16], and human–computer interaction method for making decisions [17,18,19]. Furthermore, eye movement provides a new perspective and experimental method for cognitive research [20,21,22].

Thus, there is an increasingly urgent need for quantitative comparison of eye movement indicators [23]. The scanpath map [24,25,26,27], heat map [28,29], and transition matrix [30] are several important methods for analyzing the sequence of fixation. The scanpath map represents the fixations as a sequential sequence, and vector- and character-based editing methods have been applied to calculate the similarity and difference of scanpaths. The heat map represents the eye movement data as a Gaussian mixture model, but because this method loses the sequence information of the fixations, the index based on the heat map can only reflect the similarity of different regions of the observed image, and ignores the order of fixation.

Compared with heat map, modeling the gaze transitions as a first-order Markov chain transition matrix between areas of interest (AOIs) preserves the gaze switch information. Thus, quantitative analysis based on transition matrix, of which gaze entropy (the entropy of the Markov chain) is one of the most important measures, has been used in recent years. Gaze entropy was first applied into flight simulation in [31], although it is only in recent years that it has gained a growing interest from researchers. Shiferaw et al. have recently reviewed and discussed gaze entropy in [32].

As a first-order Markov chain can be interpreted as an information channel, we proposed for the first time the gaze information channel in [33], and applied it to study the artwork of Van Gogh. In addition to incorporating the stationary entropy and gaze transition entropy, the gaze information channel paradigm allows for additional information-theoretic measures to analyze the gaze behavior. The new informational measures include joint entropy, mutual information, and normalized mutual information. The gaze channel was further explored in [34], where the scientific posters cognition was studied from the perspective of the gaze channel.

In this paper, we expand our previous work in [33,34] in several lines:

- Differently to the authors of [33,34], the gaze channel does not depend on the gaze sequences being interpreted as a first-order Markov chain.
- We study images (artworks from Van Gogh) as in [33], versus posters containing text plus images in [34].
- We study 12 Van Gogh artworks versus only three artworks, and 10 observers versus three observers in [33].
- We use nine AOIs, versus only three in [33] and up to six in [34].
- We use regular grid division into AOIs, against predetermined in [34].
- We compare vertical division vs. horizontal division, allowing us an intuitive explanation of mutual information.
- We present and interpret the evolution of gaze channel quantities with observation time.
- We compare and relate our results with informational aesthetics measures described in the literature.

The rest of the paper is organized as follows. In Section 2, we present previous work on eye tracking data analysis based on the transition matrices, in Section 3 we model the gaze sequences between AOIs as an information channel, in Section 4 and Section 5 we show experimental design and results analysis, and conclusions and future work are presented in Section 6.

## 2. Background

Vandeberg et al. [35] used a multi-level Markov modeling approach to analyse gaze switch patterns. After modeling the individuals’ gaze as Markov chains, Krejtz et al. [36,37] calculated the entropy of the stationary distribution $H_{s}$ and the transition or conditional entropy $H_{t}$ to interpret the overall distribution of attention over AOIs, as the Markov chain transition probability matrix has a dual interpretation as a conditional probability matrix. Raptis et al. [38] asked the participants to complete recognition tasks with various complexities, then the researchers used $H_{s}$ and $H_{t}$ to eye tracking analysis; the result revealed there are quantitative differences on visual search patterns among individuals. Raptis et al. [38] stated that eye gaze, including gaze entropies, fixation duration, and number, can reflect personal differences in cognitive styles.

Zhong et al. [39] modeled the relationship between the image feature and the saliency as a Markov chain, and in order to predict the transition probabilities of the Markov chain, they trained a support vector regression (SVR) from true eye tracking data. At last, when given the stationary distribution of this chain, a saliency map of predicting user’s attention can be obtained.

Huang [40] used the female gaze data of browsing apparel retailers’ web pages to study how the female attention was influenced by visual content composition and slot position in personalized banner ads. Gu et al. [30] used heatmap entropy (visual attention entropy (VAE)) and its improved version, relative VAE (rVAE) to analyze eye tracking data of observing web pages; the result showed that VAE and rVAE have correlation with the perceived aesthetics. Hwang et al. [41] stated that it is important to notice scenes consist of objects representing not only low-level visual information, but also higher-level semantic data, and they presented transitional semantic guidance computation to estimate gaze transition.

Ma et al. [33] introduced the gaze information channel using Van Gogh paintings, and, based on preliminary results, observed that we can give a coherent interpretation to the channel quantities to both classify the observers and the artworks. Hao et al. [34] tracked observers’ eye movements for reading scientific posters, which contain both text and data, and modeled eye tracking fixation sequences between AOIs as a Markov chain and subsequently as an information channel to find quantitative links between eye movements and cognitive comprehension. The AOIs were determined by the design of the poster.

## 3. Methodology

### 3.1. Gaze Information Channel

Given an image *I*, divided it into *s* AOIs, where the set of AOIs is S=1,2,…,s, let us build a matrix *C* of successively visited AOIs. Thus, element ij in matrix *C*, $c_{ij}$, will correspond to how many times the AOI *j* has been visited immediately after AOI *i* was visited, that is, how many times there has been a direct transition from *i* to *j*. This information is extracted from the recorded gaze sequences. Observe that $\sum_{j}c_{ji}$ gives the total number of times AOI *i* was visited. Observe also that if we consider an additional fictional AOI, let us say AOI number “0”, that represents both the initial state before our gaze lands on the painting and the final state when our gaze leaves the painting, then the number of exits and number of entries on any state have to be the same, this is $\sum_{j}c_{ij}=\sum_{j}c_{ji}$ for all *i* and *j*. If the trajectories are not short, $\sum_{j}c_{ij}\approx \sum_{j}c_{ji}$, for practical purposes we can consider them equal and ignore AOI “0”. Observe that matrix *C* can be considered as the realization of a joint occurrence of random variables *X* and *Y*, (X,Y), where each pair (x,y) represents the occurrence of the gaze entering AOI *x* and leaving for AOI *y*. Let $N=\sum_{i}\sum_{j}c_{ij}$, $N_{i}=\sum_{j}c_{ij}$, and $N_{j}=\sum_{i}c_{ij}$, then the joint probabilities can be constructed as $p\left(i,j\right)=c_{ij}/N$, the conditional probabilities matrix *P* as $p_{ij}=p\left(j|i\right)=c_{ij}/N_{i}$, and the marginal probabilities p(X)=p(Y), as $p_{i}=N_{i}/N$. Observe that by construction p(X)P=p(Y). We have thus built an information channel [42] between the *S* areas of interest to itself. Observe that this information channel can be considered too as a first-order Markov chain with equilibrium distribution π=p(X)=p(Y) and transition matrix *P*. In our previous work [33,34], we introduced the gaze information channel from the first-order Markov chain, while here we introduce first the information channel. The difference is not trivial, as when directly introducing the information channel we do not mind whether the gaze sequences follow a first-order or a higher-order Markov chain. However, even if the gaze does follow a higher order than first-order Markov chain, it is still possible by what we have shown before to model gaze sequences as a first-order Markov chain. In that case, the transition probabilities between states should be understood as the average ones. Given AOI *i*, $p_{ij}$ would give then the average transition probability to AOI *j*, as the transition probabilities would depend on the given instant of the total observation time, and might change from the first seconds of observation to later seconds. Previous work has considered the gaze transitions as a first-order Markov chain [32].

According to the strategy of dividing into AOIs, there are mainly content-dependent AOIs and grid AOIs. In this paper, grid AOIs are used.

### 3.2. Gaze Information Channel Measures

In this section, Shannon’s information measures [42] for gaze information channel are introduced. In addition to the gaze stationary entropy $H_{s}$ and gaze transition entropy $H_{t}$ used in previous work [36], the gaze information channel makes it possible to introduce more informational measures to study the eye movement data. In the information channel, the stationary entropy $H_{s}$ is defined as

(1) $$H_{s}=H\left(X\right)=H\left(Y\right)=-\sum_{i=1}^{s}\pi_{i}\log\pi_{i},$$

and gives the uncertainty of the distribution of the gaze between the AOIs.

The entropy of ith row, H(Y|i), is defined as

(2) $$H\left(Y|i\right)=-\sum_{j=1}^{s}p_{ij}\log p_{ij},$$

and gives the uncertainty about the next AOI when the current gaze location is the *i*-th AOI.

The conditional entropy $H_{t}$ of the information channel is given by the weighted average values of H(Y|i),

(3) $$\begin{array}{c} H_{t}=H\left(Y|X\right)=\sum_{i=1}^{s}\pi_{i}H\left(Y|i\right)=-\sum_{i=1}^{s}\pi_{i}\sum_{j=1}^{s}p_{ij}\log p_{ij}, \end{array}$$

and represents the randomness or uncertainty of next gaze transition for all AOIs.

The joint entropy H(X,Y) of the information channel is the entropy of the joint distribution of *X* and *Y*,

(4) $$\begin{array}{c} H\left(X,Y\right)=H\left(X\right)+H\left(Y|X\right)=H_{s}+H_{t}=\sum_{i=1}^{s}\sum_{j=1}^{s}\pi_{i}p_{ij}\log\left(\pi_{i}p_{ij}\right), \end{array}$$

and measures the total uncertainty of the information channel. Observe that, being for the gaze information channel p(X)=p(Y)=π, then H(X)=H(Y), and as H(X,Y)=H(Y,X) then H(Y|X)=H(X|Y).

The mutual information I(X;Y), given by

(5) $$\begin{array}{c} I\left(X;Y\right)=H\left(X\right)+H\left(Y\right)-H\left(X,Y\right)=\sum_{i=1}^{s}\sum_{j=1}^{s}\pi_{i}p_{ij}\log\frac{p_{ij}}{\pi_{j}}, \end{array}$$

indicates the total correlation, or information shared, between the AOIs.

**Figure 1:**
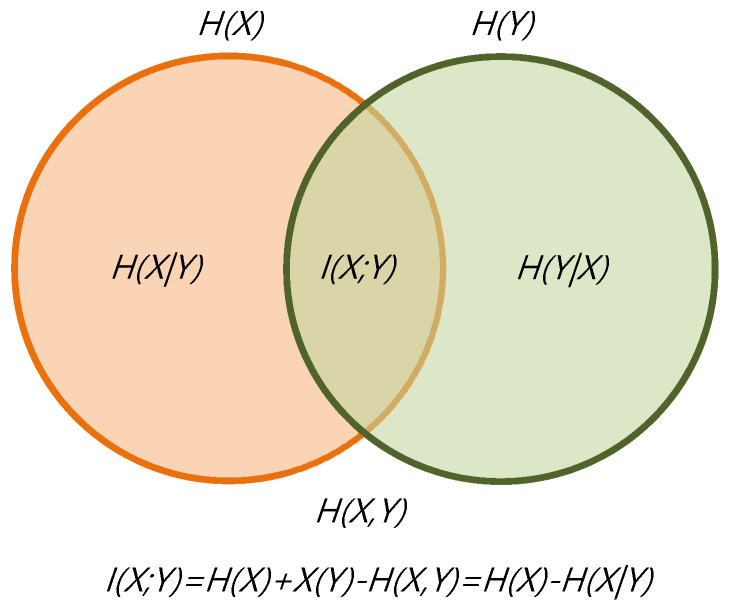
The information diagram represents the relationship between information channel measures.

The relationship between information measures can be illustrated by a Venn diagram, as shown in Figure 1. The diagram represents the relationship between Shannon’s information measures.

### 3.3. Informational Aesthetics Measures

To study the evolution of Van Gogh’s style, Jaume Rigau et al. [43,44,45,46,47] used a quantitative approach based on aesthetic measures, including palette-based relative redundancy $M_{b}$, Kolmogorov complexity-based redundancy $M_{k}$, and the number of regions for a given ratio of mutual information $M_{s}$.

Given a color image of *N* pixels, where *C* represents the palette distribution ($X_{rgb}$ with $256^{3}=2^{24}$ colors or $X_{l}$ with $256=2^{8}$ luminance values), the palette entropy H(C) stands for the uncertainty of a pixel, and the maximum entropy $H_{max}$ is 24 ($X_{rgb}$) and 8 ($X_{l}$), respectively. The relative redundancy $M_{b}$ is defined as

(6) $$M_{b}=\frac{H_{max}-H\left(C\right)}{H_{max}},$$

where $M_{b}$ ranges in [0, 1] and represents the reduction of pixel uncertainty due to the choice of a palette with a given color probability distribution instead of a uniform distribution. Observe that $M_{b}$ is similar to the redundancy per character of a natural language [48] and corresponds to Bense’s information theoretic interpretation [49] of Birkhoff’s aesthetic measure [50].

From the perspective of Kolmogorov complexity, an image’s order or regularity can be measured by the difference between the image size $N\times H_{max}$ and its Kolmogorov complexity K(I). The normalization of the order gives us the aesthetic measure

(7) $$M_{k}=\frac{N\times H_{max}-K\left(I\right)}{N\times H_{max}}$$

where $M_{k}$ ranges in [0, 1] and represents the degree of the order of the image without any prior knowledge of the palette. Note that the higher the order of the image, the higher the compression ratio.

We can segment an image into regions. The coarsest segmentation is to consider the whole image as a single region, and the finest segmentation would be to consider as many segments as pixels in the image. Given a segmentation, we represent by *R* the normalized areas of the regions. A given region can contain pixels of different colors from the palette *C*. Thus, an information channel between colors *C* and regions *R* can be established. The mutual information between *C* and *R* is given by

(8) $$I\left(C,R\right)=\sum_{c\in C}\sum_{r\in R}p\left(c,r\right)\log\frac{p(c,r)}{p(c)p(r)}$$

For a decomposition of an image into *n* regions, the ratio of mutual information is defined by

(9) $$M_{s}\left(n\right)=\frac{I(C,R)}{H(C)},$$

and ranges from 0 to 1. When we have one single region the mutual information is 0, and thus the ratio is 0. When we have as many regions as pixels we have captured the whole correlation of the image, I(C,R)=H(C) and the ratio is 1. We are interested in how many segments *n* we need to divide the single image to arrive at a given percentage of mutual information. This is given by the inverse function

(10) $$M_{s}^{-1}\left(\frac{I(C,R)}{H(C)}\right)=n,$$

and is interpreted as a measure of image compositional complexity.

In addition to the above measures, Sigaki et al. [51] presented a quantitative analysis of art by estimating the permutation entropy and the statistical complexity of a painting, considered, as in the above measures, as an array of pixel values. Given a Nx×Ny image as a two-dimensional array, subarrays of size dx×dy are considered as a single sequence of dx×dy components, and the possible order of the values of each sequence is classified into one of the n=(dxdy)! possible orderings. For instance, for dx=dy=2 we have 4!=16 possible orderings. All possible, overlapping, dx×dy subarrays are considered, and finally after normalization we will have a distribution *P* which represents the order of neighbor pixel values in the image, $P=\{p_{i};i=1,\ldots ,n\}$. The only parameters of the method are the dx,dy values, also called embedding dimensions (for a more formal description, please refer to the work in [51,52]).

Then, the normalized permutation entropy PE is calculated by dividing the Shannon entropy S(P) of *P* distribution,

(11) $$S\left(P\right)=-\sum_{i=1}^{n}p_{i}\log p_{i},$$

by its maximum possible value log(n),

(12) $$PE\left(P\right)=\frac{S(P)}{\log(n)}$$

Sigaki et al. [51] argue that although the value of PE is a good measure of randomness, it cannot fully capture the degree of structural complexity present in the image matrix. Therefore, they further calculated the so-called statistical complexity C(P)

(13) $$C\left(P\right)=\frac{Q(P,U)PE(P)}{Q_{max}}$$

where Q(P,U) is a relative entropic measure (the Jensen–Shannon divergence) between $P=\{p_{i};i=1,\ldots ,n\}$ and the uniform distribution $U=\{u_{i}=1/n;i=1,\ldots ,n\}$, and computed as

(14) $$Q\left(P,U\right)=S\left(\frac{P+U}{2}\right)-\frac{S(P)}{2}-\frac{S(U)}{2}$$

where $\frac{P+U}{2}=\left\{\frac{p_{i}+1/n}{2},i=1,\ldots ,n\right\}$ and

(15) $$Q_{max}=-\frac{1}{2}\left\{\frac{n+1}{n}\log\left(n+1\right)+\log\left(n\right)-2\log\left(2n\right)\right\}$$

is a normalization constant obtained by calculating Q(P,U).

## 4. Experimental Design

### 4.1. Participants

Twelve Master’s students from Tianjin University were selected to take part in the experiment. All participants had normal or corrected-to-normal vision. Twenty minutes before the start of the experiment, all participants were forbidden to play on mobile phones or perform reading activities that may cause visual fatigue, and to perform eye exercises, such as activities that can relax the eyes and mind and body. The data from two participants had to be excluded because their eye tracking rate was below 98%. Finally, eye movement data of 10 students (6 females, 4 males, average age 24.8) were available for the study.

### 4.2. Stimuli

**Figure 2:**
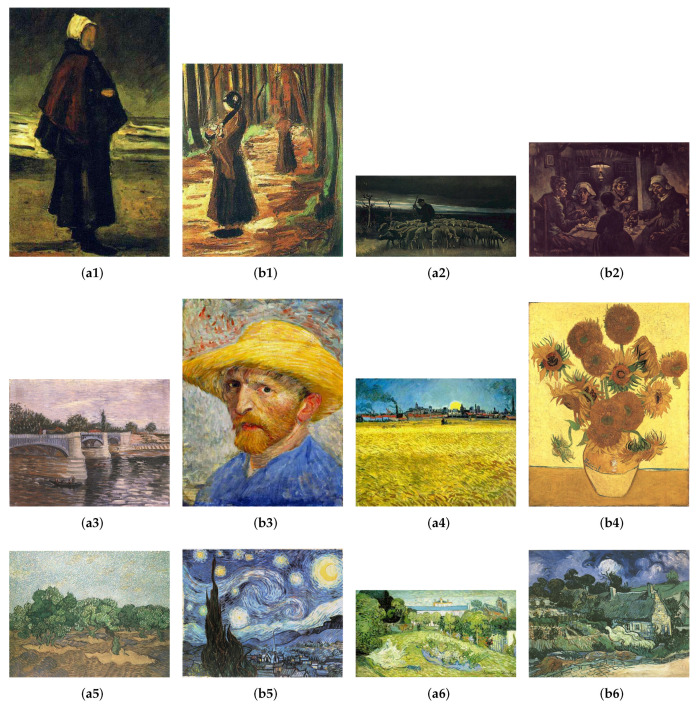
Group a and b of representative paintings of each period are shown (chronologically ordered from period 1:(a1&b1) to period 6:(a6&b6), Copyright 1996–2010 DavidBrooks). The values of $M_{B},M_{K},$ and $M_{s}^{-1}\left(0.25\right)$ are labeled for each painting. (**a1**) Fisherman’s Wife on the Beach, 1882 (0.418, 0.759, 1264). (**b1**) Two women in the Woods, 1882 (0.310, 0.650, 2020). (**a2**) Shepherd with a Flock of Sheep, 1884 (0.463, 0.739, 875). (**b2**) The Potato Eaters, 1885 (0575, 0.850, 1417). (**a3**) The Seine with the Pont de la Grande Jette, 1887 (0.385, 0.718, 1396). (**b3**) Self-Portrait with Straw Hat, 1887 (0.295, 0.726, 1272). (**a4**) Sunset: Wheat Fields Near Arles, 1888 (0.345, 0.697, 1648). (**b4**) Vase with Fifteen Sunflowers, 1888 (0.349, 0.581, 2736). (**a5**) Olive Grove: Pale Blue Sky, 1889 (0.339, 0.593, 2456). (**b5**) Starry Night, 1889 (0.322, 0.594, 1758). (**a6**) Daubigny’s Garden, 1890 (0.315, 0.714, 2375). (**b6**) Thatched Cottages at Cordeville, 1890 (0.312, 0.592, 2095).

The stimuli are 12 paintings of Vincent Van Gogh in digital format. Van Gogh’s paintings are classified into six periods, which follow chronological order, as Earliest Paintings (1881–1883), Nuenen/Antwerp (1883–1886), Paris (1886–1888), Arles (1888–1889), Saint-Remy (1889–1890), and Auvers-sur-Oise (1890), respectively. The paintings are divided in two groups (a and b) as shown in Figure 2. Both groups include 6 representative paintings of each period (periods numbered from 1 to 6). We have considered the two groups of paintings used by Feixas et al. [53], which gives the values of the measures ($M_{b},M_{k},M_{s}$) for the 12 paintings. The 12 paintings were downloaded from The Vincent Van Gogh Gallery of David Brooks, http://www.Vggallery.com, a website remaining the most thorough and comprehensive Van Gogh resource on the World Wide Web.

### 4.3. Apparatus

The experiment used a mobile eye tracking device SMIETG2w produced by the German SMI company. This eye tracker’s two non-contact infrared cameras (60/120 Hz) can capture images of the observer’s eyes, and calculate eye movements in real-time based on the pupil and corneal reflection principles. Another camera of the eye tracker can record image scene that the observer is viewing. In addition, the eye tracker is also equipped with a USB cable to transfer the data collected by the camera to the eye tracking control system.

The eye tracking control system is a high-performance workstation installed with IView X software. The video data collected by the eye movement instrument is integrated into the workstation for image data analysis after MPEG coding. Eye movement data acquisition software IView X can complete the fixation point calibration before formal observation. In our work, we adopted three-point calibration with higher accuracy. After the data collection is completed, the Begaze software can be used to generate fixation position.

### 4.4. Procedure

The calibration picture and the 12 Van Gogh paintings used in the formal experiment were presented on a computer monitor (1920×1080 resolution; 23.8-inch LCD). The participant was invited to sit in a chair in front of the monitor, their eyes about 60 to 80 cm away from the screen, and chin resting on a fixed bracket. Then, the staff used the IView X software to make 3-point calibration for each participant. After calibration, the Van Gogh paintings were displayed in full screen, in random order. The observation time of each painting is 45 s, there are 10 s for rest after each painting is displayed, and the viewing mode is free-viewing, that is, no viewing task is assigned to the observer. Before the observation, the researcher does not disclose any information about the painting to be observed to the participant, which aims to reduce the influence of top-down factors and facilitate the analysis of the relationship between human eye behavior and the painting content itself.

## 5. Result Analysis

### 5.1. Channel Measures Analysis with 9 AOIs

**Figure 3:**
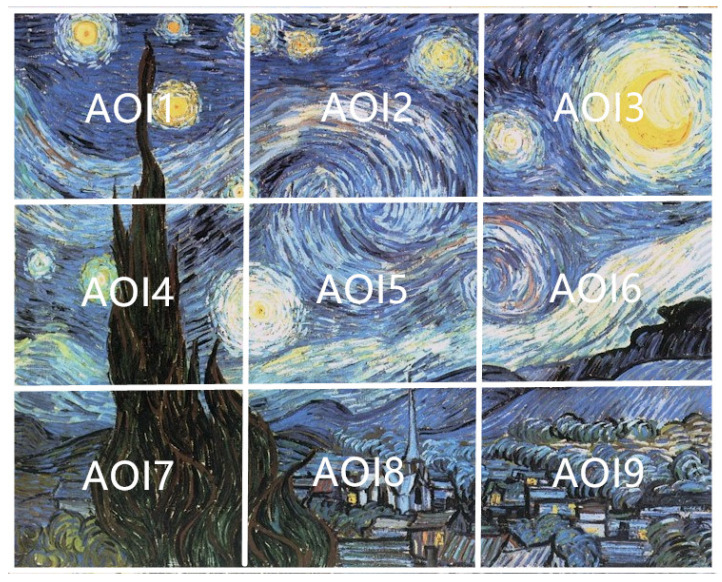
An example painting divided in the 9 AOIs.

**Figure 4:**
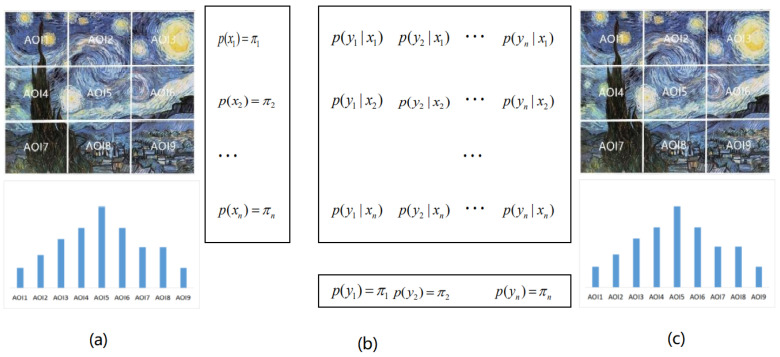
The gaze information channel for painting b5 with 9 AOIs, between the AOIs with equilibrium distribution (**a**,**c**) and information channel X→Y (**b**). Observe that the input (**a**) and output (**c**) distributions are the same.

**Figure 5:**
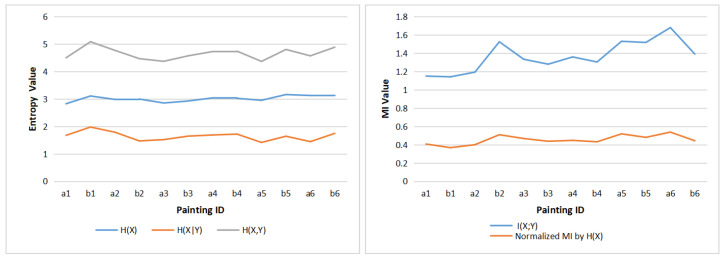
The clustered entropies (**left**) and clustered MI values (**right**) of 12 paintings.

Each painting was divided into nine AOIs (as shown in Figure 3). This number of AOIs is a compromise between the detail we look in the analysis and the sparseness of the transition matrices. In order to demonstrate the differences when observing each painting, for each AOI, we add the fixations of the 10 observers together, then we use the gaze information channel (as shown in Figure 4) to compute the clustered entropy and MI for each painting. The clustered values for all observers are shown in the Appendix A in Table A1, Table A2, Table A3, Table A4, Table A5, Table A6, Table A7, Table A8, Table A9, Table A10, Table A11 and Table A12. We have built the equilibrium distribution π=p(X)≈p(Y) by normalizing the row totals. Table 1 shows the values of entropy, MI, normalized MI, and the aesthetics measures from Section 3.3 for the 12 paintings. The validity of the clustering strategy was shown in [34]. From Figure 5 left, we can observe that there is little variation of H(X), while there is an important variation of H(X,Y) values, mainly due to the variation of H(X|Y) (as H(X,Y)=H(X)+H(X|Y)). The values of H(X|Y) have a tendency to decrease from left to right, according to the chronological order of the paintings. From Figure 5 right we observe an increase in mutual information, attenuated in the case of normalized one. Remembering that paintings are ordered according to the evolution in the time of Van Gogh styles, and the interpretation of H(X|Y) as randomness and of I(X,Y) as correlation between the AOIs of the painting, Van Gogh style evolution towards its maturity, with richer compositions, is reflected in an increase of mutual information in the gaze channel.

**Figure 6:**
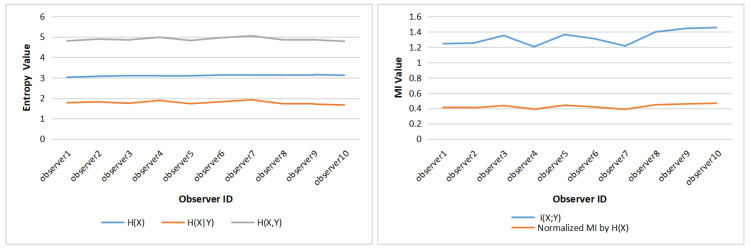
The clustered entropies (**left**) and clustered MI values (**right**) for 10 observers.

Next, in order to study the individual differences between observers, the clustered entropy and MI for each observer are computed: for each AOI, we add the fixations of 12 paintings together, then use the gaze information channel compute the clustered entropy and MI for each painting. Table 2 shows the clustered entropy and MI for 10 observers, and Figure 6 shows the clustered entropies and MI.

Comparing Figure 6 with Figure 5, it can be inferred that there is not as much difference between observers as there is between paintings. In Table 2, similar to Table 1, the standard deviation of H(X) is the lowest among H(X), H(X|Y) and H(X,Y) values, thus the differences happen more in gaze switch among AOIs (given by H(X|Y)) than in the attention distribution among AOIs (given by H(X)). Moreover, from Figure 6 left, it can be observed that the H(X), H(X|Y) and H(X,Y) present close values for the different observers. Figure 6 right shows that the main differences between observers can be found in the values of mutual information, with basically two kind of observers: ones with lower MI, around 1.2, and the other ones with higher MI, around 1.4. These differences are smoothed down when considering normalized MI.

### 5.2. Comparison of Horizontal with Vertical Division

**Figure 7:**
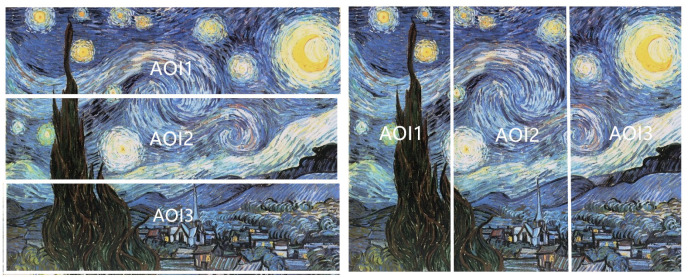
An example of horizontal division (**left**) and vertical division (**right**) with 3 AOIs.

When reading text, human eye movement behavior is greatly affected by the direction of text layout. For example, if the text is arranged as usually in horizontal lines, our eyes will move in horizontal direction during reading. However, the direction of eye movement is more unpredictable when viewing images or paintings. Because of the differences in the content of the paintings, the observer’s attention distribution in different areas will be different. Therefore, in order to study the characteristics of the observer’s gaze switch and attention distribution, according to a different division into AOIs, we compared gaze entropy and mutual information values obtained in horizontal and vertical divisions into three AOIs, see Figure 7.

As done for nine AOIs, the gaze sequences of the 12 paintings are integrated, and gaze channel measures of each observer are calculated under horizontal and vertical divisions, respectively, to analyze the characteristics of eye movement behavior of each observer. Similarly, to obtain the gaze measures for each painting, the gaze sequences of the 10 observers are firstly integrated, and then processed with the gaze information channel based on horizontal and vertical division AOIs.

**Figure 8:**
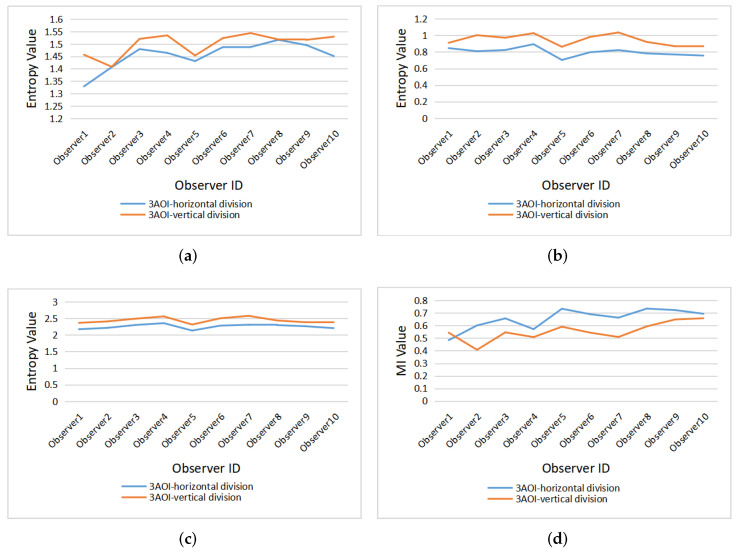
The clustered gaze measures for all observers under horizontal and vertical division: (**a**) H(X), (**b**) H(X|Y), (**c**) H(X,Y), (**d**) I(X;Y).

Figure 8 gives the clustered gaze information measures H(X), H(X|Y), H(X,Y), and I(X;Y) from all observers under the horizontal and vertical division. For the vertical division, the entropies (H(X), H(X|Y) and H(X,Y)) are higher than the entropy measures of horizontal division, while the mutual information I(X;Y) from observers is lower in general under the vertical division, except for observer1. The larger mutual information represents the stronger relevance of the gaze in the area of horizontal division, so it can be concluded that gaze shift is more likely to occur in the horizontal direction.

**Figure 9:**
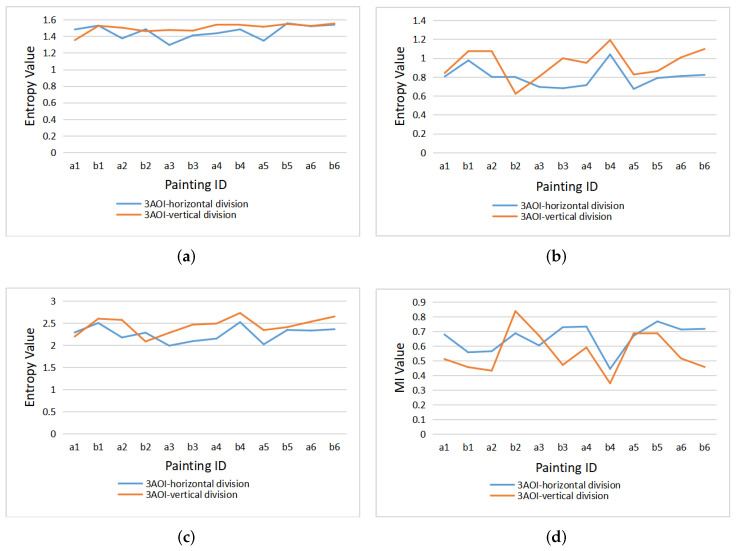
The clustered gaze measures for all paintings under horizontal and vertical division: (**a**) H(X), (**b**) H(X|Y), (**c**) H(X,Y), (**d**) I(X;Y).

Figure 9 presents the clustered gaze information measures H(X), H(X|Y), H(X,Y), and I(X;Y) of the 12 paintings under horizontal and vertical division. Similar to Figure 8, for most paintings, the gaze measures H(X), H(X|Y), and H(X,Y) generated by vertical division are higher than for horizontal division, while the mutual information is lower than for horizontal division. However, painting a1 and painting b2 do not follow this rule, as the H(X) and H(X,Y) of painting a1 are higher in horizontal than vertical division, and the difference for H(X|Y) of painting a1 between horizontal and vertical division is the smallest of all paintings. On the other hand, for painting b2, all four measures (H(X), H(X|Y), H(X,Y), and I(X;Y)) have the opposite rules than for the other paintings.

For the difference of gaze measures caused by the two division types in Figure 8 and Figure 9, we can forward the following explanation; on the one hand, the difference between the results of horizontal and vertical division is related to people’s inherent reading mode, and thus when the area of interest is divided horizontally, the number of gaze switches between different AOIs is relatively small, that is, the H(X|Y) value is low. On the other hand, the painting content also has an important impact on the eye movement mode. For paintings a2 and a4 (as shown in Figure 2), the horizontal division splits coherently the sky and the field, with higher mutual information, while the vertical line cuts off the continuous scene, resulting in higher entropy measures and lower mutual information. For paintings a1 and b2 (as shown in Figure 2), the main body of the picture is the person. In the process of observation, people tend to observe the coherent content continuously, so the gaze shift occurs more in the vertical direction.

### 5.3. Comparison with Different Varying Observation Time

**Figure 10:**
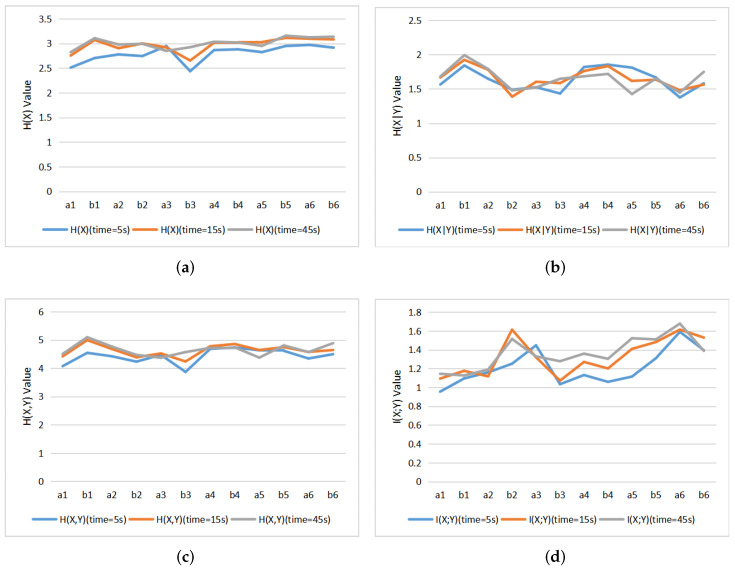
The line charts of 12 paintings with entropies and MI of different observation time: (**a**) H(X), (**b**) H(X|Y), (**c**) H(X,Y), (**d**) I(X;Y).

Figure 10 are line charts of 12 paintings with entropies and MI for different observation time. It can be seen that the H(X), H(X,Y), and I(X;Y) basically present an increasing trend until a stable value is reached. First, the global scanning of image over the time is illustrated by the change of H(X), which gradually increases and tends to be stable, indicating that the distribution of fixation points are more evenly between the different AOIs. This increase in H(X) pushes the increase of H(X,Y). On the other hand, the increase in I(X;Y) tends to correspond to a decrease in H(X|Y). The more we explore the image, the more correlation, or mutual information, we can discover, and the less the uncertainty in exploration, given by H(X|Y). We could thus divide observation behavior into two stages. In a first stage the observer will scan the image globally, without a specific aim or plan, and after that, the observer will focus on more details and in correlations within the image. This fits with the observations by Locher et al. [54,55].

### 5.4. Comparison with Aesthetic Measures

In this section, we study the relationship of gaze channel measures with the information aesthetics measures from Section 3.3. In Table 1, the values of entropy, MI, $M_{k}$, $M_{b}$, and $M_{s}$ are given for the 12 paintings.

#### 5.4.1. Comparison with $M_{b}$

**Figure 11:**
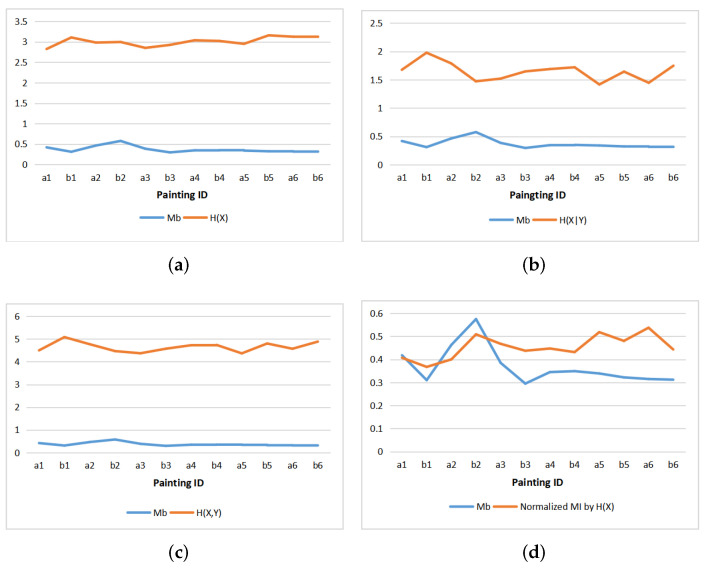
Comparing $M_{b}$ with entropies and normalized mutual information of paintings: (**a**) $M_{b}$ and H(X), (**b**) $M_{b}$ and H(X|Y), (**c**) $M_{b}$ and H(X,Y), (**d**) $M_{b}$ and normalized MI.

Figure 11 shows the line charts of $M_{b}$ and entropies and normalized MI for the 12 paintings. We see that the behavior of $M_{b}$ is rather opposite to the behavior of entropies, and presents some similarity with normalized mutual information. This can be interpreted as the measure $M_{b}$ representing correlation or redundancy in the scene. In fact, from its definition, $M_{b}$ is the normalized difference between the maximum entropy of the color histogram and the color histogram used in the painting, and thus represents the redundancy existing in the palette used, giving a certain measure of correlation. However, $M_{b}$ does not take into account any spacial order, thus we can not expect any accurate correlation.

#### 5.4.2. Comparison with $M_{k}$

**Figure 12:**
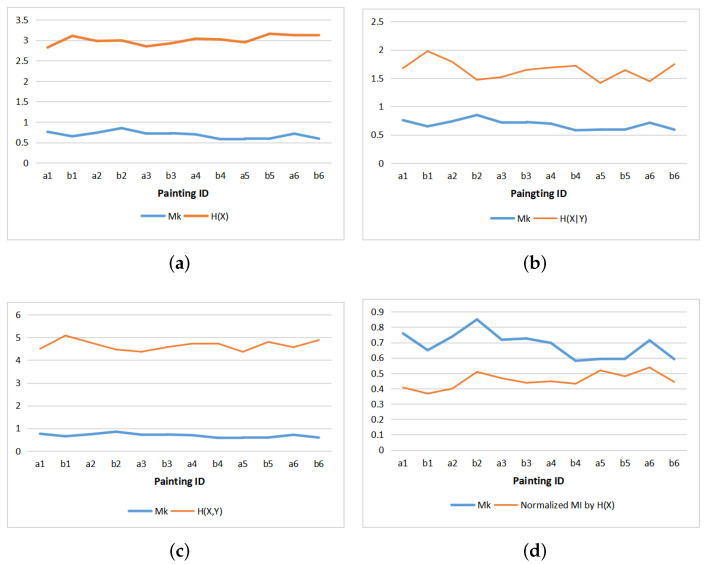
Comparing $M_{k}$ with entropies and normalized mutual information of paintings: (**a**) $M_{k}$ and H(X), (**b**) $M_{k}$ and H(X|Y), (**c**) $M_{k}$ and H(X,Y), (**d**) $M_{k}$ and normalized MI.

From information theory perspective, the positive correlation shown in Figure 12d between normalized MI and $M_{k}$ can be explained by the theoretical correspondence or similarity between the entropy rate expressed by H(Y/X) and the Kolmogorov complexity K(I) approximated by the file length of the compressed image. Let us remember that $M_{K}$ is given by $1-\frac{K(I)}{N\times H_{max}}$ and normalized MI by $1-\frac{I(X;Y)}{H(X)}$. Thus, instead of analyzing the correlation between normalized MI and $M_{k}$, we can equivalently analyze the relationship between the entropy rate and the Kolmogorov complexity.

Both measures express, from two different perspectives, the notion of compression. On the one hand, the entropy rate H(Y/X) of a communication process quantifies the irreducible randomness in sequences produced by a source and also measures the size, in bits per symbol, of the optimal binary compression of the source [56]. Thus, a process highly random is difficult to compress. On the other hand, as mentioned above, the Kolmogorov complexity represents the difficulty in compressing an image, expressed by a set of bits which describes both its regularities and its random part. In our case, these measures are normalized in order to carry out a comparative study.

**Figure 13:**
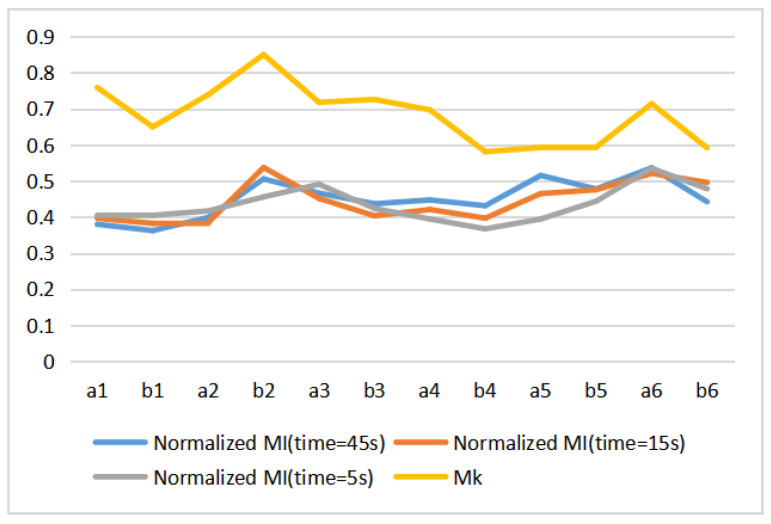
Comparison $M_{k}$ and normalized MI by H(X) at different observation times.

We visualize also in Figure 13 the change of normalized mutual information versus the increase in viewing time from 5 s, 15 s, to 45 s, the value of gaze mutual information will also change.

#### 5.4.3. Relationship with $M_{s}^{-1}$

We can find an indirect relationship of gaze information channel measures with $M_{s}^{-1}$. If we look in [53] at the spatial division triggered by the color to region channel, we can see that the first divisions, which give the maximum increase in mutual information, are triggered along horizontal divisions. This is fully in concordance with the findings in Section 5.2.

#### 5.4.4. Comparison with PE and *C*

**Figure 14:**
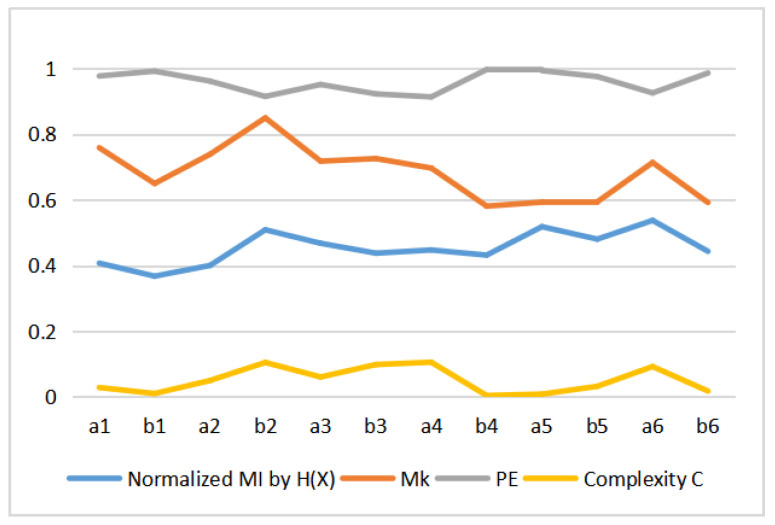
Comparison of normalized MI by H(X), $M_{k}$, permutation entropy PE and complexity *C*.

**Figure 15:**
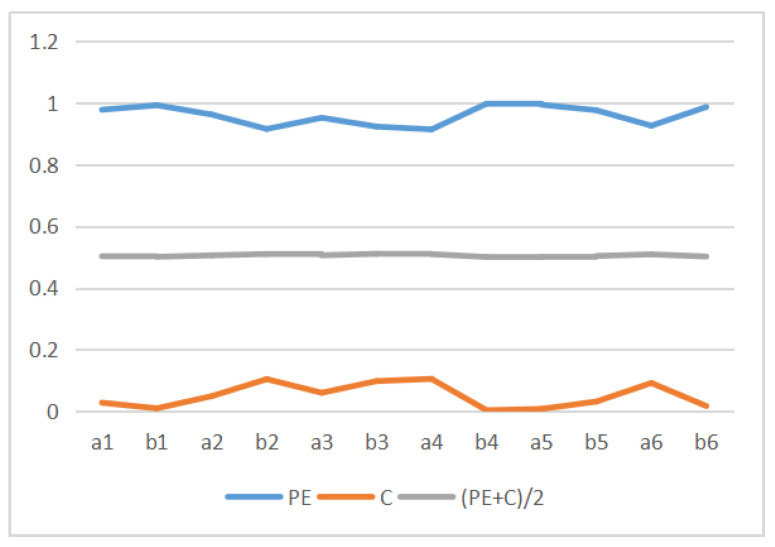
Illustration of the dependency of *C* vs PE for the values corresponding to the 12 paintings.

**Figure 16:**
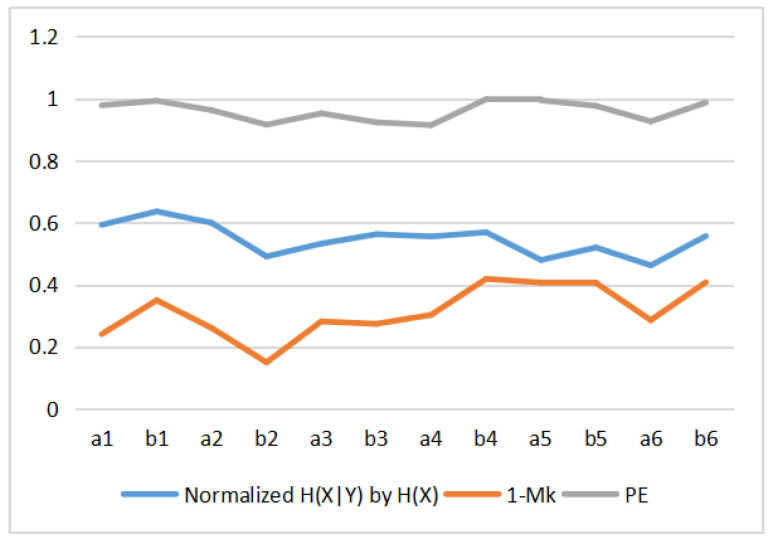
Comparing normalized H(X|Y) by H(X), $1-M_{k}$ and permutation entropy PE of paintings.

Table 3 shows the values of MI, $M_{k}$, permutation entropy PE, and complexity *C* for the 12 Van Gogh paintings considered. These values are displayed in Figure 14. We can observe that the MI, $M_{k}$ and complexity *C* have similar curves pattern, but permutation entropy PE behavior is different. In fact, we can observe that *C* behavior is C≈1−PE, see Figure 15. This can be explained as follows. The behavior of the normalized Jensen–Shannon distance Q(P;U) is similar to 1−PE, and thus *C*, defined by Equation (13), can be approximated by C≈(1−PE)PE, and for values of PE near 1 as it is our case C≈(1−PE). This is illustrated in Figure 15. We display in Figure 16 the normalized H(X|Y) (normalized H(X|Y) + normalized I(X,Y) =1), $1-M_{k}$ and PE. The correspondence between normalized H(X|Y) and $1-M_{k}$ is the same as between normalized I(X,Y) and $M_{k}$. However, observe now the correspondence with PE too. Correspondence between the Kolmogorov complexity measured by compressibility, $1-M_{k}$, and the normalized permutation entropy, PE was somehow to be expected, as PE measures [51], the degree of disorder in the pixel arrangement of an image, and the more disorder we can expect less compressibility and bigger size of compressed file, and vice versa.

## 6. Conclusions and Future Work

This paper uses quantitative indicators based on gaze information channel to study the relationship between Van Gogh artworks and human viewing. The eye tracking fixation sequences through areas of interest (AOIs) are modeled as an information channel, which extends the Markov chain modeling of those sequences.

For our study, we have used 12 Van Gogh paintings, two from each of the six periods in which critics classify Van Gogh art work. We have first shown that, with nine AOIs, the measures discriminate better between the different paintings than between the different observers. Then we have compared the values obtained with horizontal and vertical division into three AOIs, and found that in general the mutual information is higher for horizontal division. This can be put in correspondence with the semantic content of the painting.

Finally, we have compared previously defined computational measures to study artworks with the measures derived from the information channel paradigm. We have shown the relationship between the computational measures, which are independent of any observer, and the information channel measures, which come from the eye trajectories from human observers. In particular we have found a striking visual correlation between the measure $M_{k}$ which is related to the compressibility and the normalized mutual information MI, and inversely, between the normalized entropy of the channel, $M_{k}$, and the permutation entropy, used recently to classify artworks.

Although promising, this paper has some limitations such as a small number of participants and paintings. With more data we could study quantitatively the correlations in addition to visually. Moreover, a larger variety of persons (e.g., laypersons vs. experts as in [57]) or painting styles (e.g., abstract vs. representational as in [58]) can be considered, as well as the aesthetic evaluation of the paintings by the observer. In the future, we will continue to explore the unique significance of human visual search patterns, which need to be paired with behavioral or cognitive metrics.

## Figures and Tables

**Table 1:** The clustered entropies, MI, $M_{k}$, $M_{b}$, and $M_{s}^{-1}$, for 12 paintings.

ID	H(X)	H(X|Y)	H(X,Y)	I(X;Y)	Normalized MI by H(X)	$M_{k}$	$M_{b}$	$M_{s}^{-1}$
a1	2.8246	1.6747	4.4993	1.1499	0.4071	0.759	0.418	1264
b1	3.1072	1.9764	5.0835	1.1417	0.3674	0.650	0.310	2020
a2	2.9821	1.7874	4.7695	1.1930	0.4000	0.739	0.463	875
b2	2.9958	1.4709	4.4667	1.5244	0.5088	0.850	0.575	1417
a3	2.8518	1.5188	4.3706	1.3336	0.4676	0.718	0.385	1396
b3	2.9251	1.6460	4.5711	1.2799	0.4375	0.726	0.295	1272
a4	3.0384	1.6874	4.7259	1.3591	0.4473	0.697	0.345	1648
b4	3.0211	1.7187	4.7399	1.3039	0.4316	0.581	0.349	2736
a5	2.9510	1.4157	4.3667	1.5296	0.5183	0.593	0.339	2456
b5	3.1591	1.6422	4.8013	1.5174	0.4803	0.594	0.322	1758
a6	3.1250	1.4451	4.5702	1.6797	0.5375	0.714	0.315	2375
b6	3.1379	1.7465	4.8844	1.3906	0.4432	0.592	0.312	2095
Average Value	3.0099	1.6441	4.6541	1.3669	0.4539	0.684	0.369	1776
Standard Deviation	0.1054	0.1547	0.2084	0.1615	0.0488	0.080	0.078	542

**Table 2:** The clustered entropies and MI for 10 observers.

ID	H(X)	H(X|Y)	H(X,Y)	I(X;Y)	Normalized MI by H(X)
observer1	3.0243	1.7807	4.8049	1.2444	0.4115
observer2	3.0720	1.8207	4.8926	1.2531	0.4079
observer3	3.1014	1.7515	4.8529	1.3514	0.4357
observer4	3.0953	1.8891	4.9844	1.2048	0.3892
observer5	3.0950	1.7306	4.8257	1.3639	0.4407
observer6	3.1359	1.8256	4.9615	1.3090	0.4174
observer7	3.1335	1.9187	5.0522	1.2155	0.3879
observer8	3.1296	1.7296	4.8592	1.3986	0.4469
observer9	3.1534	1.7098	4.8631	1.4440	0.4579
observer10	3.1200	1.6647	4.7847	1.4557	0.4666
Average Value	3.1060	1.7821	4.8881	1.3240	0.4262
Standard Deviation	0.0357	0.0767	0.0811	0.0879	0.0261

**Table 3:** The MI, $M_{k}$, permutation entropy PE and complexity *C* for 12 paintings.

ID	Normalized MI by H(X)	$M_{k}$	PE	*C*
a1	0.4071	0.759	0.9777	0.0279
b1	0.3674	0.650	0.9924	0.0097
a2	0.4000	0.739	0.9621	0.0493
b2	0.5088	0.850	0.9154	0.1044
a3	0.4676	0.718	0.9518	0.0601
b3	0.4375	0.726	0.9231	0.0981
a4	0.4473	0.697	0.9140	0.1051
b4	0.4316	0.581	0.9973	0.0036
a5	0.5183	0.593	0.9940	0.0078
b5	0.4803	0.594	0.9760	0.0316
a6	0.5375	0.714	0.9260	0.0919
b6	0.4432	0.592	0.9867	0.0172
Average Value	0.4539	0.6844	0.9597	0.0506
Standard Deviation	0.0488	0.0800	0.0311	0.0383

## Appendix A

**Table A1:** The (unnormalized) transition matrices (9×9) of painting a1.

a1	AOI1	AOI2	AOI3	AOI4	AOI5	AOI6	AOI7	AOI8	AOI9	Total
AOI1	11	6	2	5	0	0	0	0	0	24
AOI2	7	114	8	5	30	2	1	2	0	169
AOI3	2	8	12	0	3	7	0	0	0	32
AOI4	3	3	1	64	20	9	4	1	1	106
AOI5	1	27	0	16	134	15	1	23	0	217
AOI6	0	8	9	2	11	86	0	3	5	124
AOI7	0	0	0	6	1	1	28	14	0	50
AOI8	1	2	0	6	18	1	14	117	7	166
AOI9	0	0	0	1	0	4	2	7	13	27
Total	25	168	32	105	217	125	50	167	26	915

**Table A2:** The (unnormalized) transition matrices (9×9) of painting a2.

a1	AOI1	AOI2	AOI3	AOI4	AOI5	AOI6	AOI7	AOI8	AOI9	Total
AOI1	17	1	1	9	1	1	1	0	0	31
AOI2	7	12	1	2	0	10	0	1	0	33
AOI3	0	8	8	0	0	0	2	0	0	18
AOI4	4	0	1	60	10	9	0	4	0	88
AOI5	1	1	0	9	34	1	0	8	2	56
AOI6	1	6	1	10	5	73	6	12	5	119
AOI7	0	0	5	1	0	10	22	0	3	41
AOI8	0	2	0	2	4	8	2	40	11	69
AOI9	1	1	1	1	2	2	8	6	42	64
Total	31	31	18	94	56	114	41	71	63	519

**Table A3:** The (unnormalized) transition matrices (9×9) of painting a3.

a1	AOI1	AOI2	AOI3	AOI4	AOI5	AOI6	AOI7	AOI8	AOI9	Total
AOI1	45	8	0	14	3	0	0	0	0	70
AOI2	8	25	7	1	16	2	1	0	0	60
AOI3	0	6	20	0	0	4	0	0	0	30
AOI4	10	4	0	118	16	1	12	5	1	167
AOI5	3	15	1	23	186	30	3	14	1	276
AOI6	1	1	0	1	33	168	0	2	10	216
AOI7	2	0	0	7	6	1	50	16	1	83
AOI8	1	1	0	2	16	3	17	90	4	134
AOI9	0	0	2	0	0	8	0	6	22	38
Total	70	60	30	166	276	217	83	133	39	1074

**Table A4:** The (unnormalized) transition matrices (9×9) of painting a4.

a1	AOI1	AOI2	AOI3	AOI4	AOI5	AOI6	AOI7	AOI8	AOI9	Total
AOI1	115	22	2	17	2	0	4	0	1	163
AOI2	24	120	17	3	15	2	0	0	3	184
AOI3	0	22	160	3	4	5	1	0	2	197
AOI4	14	1	1	44	19	1	7	2	2	91
AOI5	3	16	5	15	74	17	2	3	2	137
AOI6	0	2	7	3	13	41	1	3	6	76
AOI7	2	1	2	3	1	1	41	12	2	65
AOI8	0	0	1	2	5	2	8	29	15	62
AOI9	0	0	1	1	4	7	2	13	48	76
Total	158	184	196	91	137	76	66	62	81	1051

**Table A5:** The (unnormalized) transition matrices (9×9) of painting a5.

a1	AOI1	AOI2	AOI3	AOI4	AOI5	AOI6	AOI7	AOI8	AOI9	Total
AOI1	22	10	0	6	3	0	2	3	3	49
AOI2	7	26	7	1	8	0	1	0	1	51
AOI3	1	7	34	0	2	6	0	0	0	50
AOI4	10	0	1	101	15	0	8	0	0	135
AOI5	2	3	0	21	139	18	4	5	8	200
AOI6	1	1	5	0	19	206	0	1	20	253
AOI7	0	2	0	4	4	1	87	13	1	112
AOI8	0	0	2	0	7	1	11	60	14	95
AOI9	5	0	0	0	3	23	0	15	73	119
Total	48	49	49	133	200	255	113	97	120	1064

**Table A6:** The (unnormalized) transition matrices (9×9) of painting a6.

a1	AOI1	AOI2	AOI3	AOI4	AOI5	AOI6	AOI7	AOI8	AOI9	Total
AOI1	73	7	4	0	1	13	0	0	0	98
AOI2	10	85	22	0	9	2	0	0	1	129
AOI3	1	29	148	9	6	13	3	3	13	225
AOI4	0	1	15	109	17	0	0	21	0	163
AOI5	1	6	2	25	111	0	0	0	1	146
AOI6	13	2	13	0	1	99	14	0	2	144
AOI7	0	1	2	1	1	16	81	0	13	115
AOI8	0	0	2	19	0	0	0	71	12	104
AOI9	0	0	17	0	0	1	16	8	91	133
Total	98	131	225	163	146	144	114	103	133	1257

**Table A7:** The (unnormalized) transition matrices (9×9) of painting b1.

a1	AOI1	AOI2	AOI3	AOI4	AOI5	AOI6	AOI7	AOI8	AOI9	Total
AOI1	88	8	18	19	3	1	2	0	3	142
AOI2	5	40	13	2	7	15	0	1	1	84
AOI3	16	15	61	1	12	3	0	0	1	109
AOI4	22	2	3	67	6	0	15	3	0	118
AOI5	4	9	10	7	54	8	0	5	0	97
AOI6	0	8	4	1	6	28	1	2	14	64
AOI7	1	0	0	13	0	2	35	16	3	70
AOI8	0	1	0	5	6	2	14	27	10	65
AOI9	1	1	0	0	3	8	3	11	34	61
Total	137	84	109	115	97	67	70	65	66	810

**Table A8:** The (unnormalized) transition matrices (9×9) of painting b2.

a1	AOI1	AOI2	AOI3	AOI4	AOI5	AOI6	AOI7	AOI8	AOI9	Total
AOI1	55	11	0	13	2	0	1	0	0	82
AOI2	12	122	8	4	19	2	0	0	1	168
AOI3	0	11	50	0	1	12	0	1	1	76
AOI4	13	3	1	123	22	1	16	6	1	186
AOI5	2	18	0	26	248	10	2	39	3	348
AOI6	0	0	15	1	12	109	0	2	18	157
AOI7	0	1	0	13	5	2	54	4	0	79
AOI8	0	0	0	6	36	2	6	155	15	220
AOI9	0	0	2	0	3	19	0	15	83	122
Total	82	166	76	186	348	157	79	222	122	1438

**Table A9:** The (unnormalized) transition matrices (9×9) of painting b3.

a1	AOI1	AOI2	AOI3	AOI4	AOI5	AOI6	AOI7	AOI8	AOI9	Total
AOI1	38	14	4	8	1	1	0	0	0	66
AOI2	11	51	18	2	5	4	0	0	0	91
AOI3	6	16	45	0	2	11	0	0	0	80
AOI4	9	2	0	51	24	0	6	0	0	92
AOI5	1	3	1	22	212	30	0	23	3	295
AOI6	0	3	12	0	28	121	1	9	7	181
AOI7	0	0	0	7	0	0	32	14	3	56
AOI8	0	0	0	3	22	3	14	70	15	127
AOI9	0	1	0	0	1	10	3	12	27	54
Total	65	90	80	93	295	180	56	128	55	1042

**Table A10:** The (unnormalized) transition matrices (9×9) of painting b4.

a1	AOI1	AOI2	AOI3	AOI4	AOI5	AOI6	AOI7	AOI8	AOI9	Total
AOI1	81	0	14	4	0	23	0	0	0	122
AOI2	1	32	6	3	0	0	0	14	3	59
AOI3	7	9	53	32	0	2	1	1	1	106
AOI4	4	7	28	179	15	17	8	17	7	282
AOI5	0	1	0	21	84	3	27	5	7	148
AOI6	23	0	2	13	2	140	21	0	0	201
AOI7	2	0	0	5	32	17	84	1	1	142
AOI8	2	9	0	22	6	0	2	99	14	154
AOI9	0	1	1	2	9	0	0	17	32	62
Total	120	59	104	281	148	202	143	154	65	1276

**Table A11:** The (unnormalized) transition matrices (9×9) of painting b5.

a1	AOI1	AOI2	AOI3	AOI4	AOI5	AOI6	AOI7	AOI8	AOI9	Total
AOI1	113	24	3	20	3	1	3	0	0	167
AOI2	27	87	21	6	13	4	1	0	0	159
AOI3	3	22	149	0	2	16	0	0	1	193
AOI4	17	3	1	71	27	2	18	0	0	139
AOI5	6	20	4	16	99	16	2	17	4	184
AOI6	0	3	13	1	17	92	0	4	14	144
AOI7	0	0	0	21	7	0	87	15	0	130
AOI8	1	0	1	5	14	3	19	105	18	166
AOI9	0	1	0	0	2	9	1	24	114	151
Total	167	160	192	140	184	143	131	165	151	1433

**Table A12:** The (unnormalized) transition matrices (9×9) of painting b6.

a1	AOI1	AOI2	AOI3	AOI4	AOI5	AOI6	AOI7	AOI8	AOI9	Total
AOI1	62	5	15	2	12	2	0	0	1	99
AOI2	4	78	17	0	3	2	0	9	0	113
AOI3	13	15	105	2	4	14	0	2	1	156
AOI4	4	1	2	75	15	1	20	3	1	122
AOI5	8	0	5	21	103	15	1	2	1	156
AOI6	4	1	9	5	15	132	12	22	3	203
AOI7	0	1	0	12	2	15	67	5	23	125
AOI8	0	9	0	3	2	17	7	86	18	142
AOI9	0	1	3	3	0	4	18	15	74	118
Total	95	111	156	123	156	202	125	144	122	1234
